# No advantage of antimicrobial prophylaxis in AML/MDS/CMML patients treated with azacitidine—a prospective multicenter study by the Polish Adult Leukemia Group

**DOI:** 10.3389/fonc.2024.1404322

**Published:** 2024-06-13

**Authors:** Krzysztof Mądry, Karol Lis, Elzbieta Sienkiewicz, Joanna Drozd-Sokołowska, Przemysław Biecek, Oktawia Sośnia, Aleksandra Gołos, Magdalena Olszewska-Szopa, Agata Obara, Zuzanna Walkowiak, Joanna Ściesińska, Edyta Subocz, Aleksandra Butrym, Rafał Machowicz, Katarzyna Budziszewska, Grzegorz Basak

**Affiliations:** ^1^ Department of Hematology, Transplantation and Internal Diseases, Medical University of Warsaw, Warsaw, Poland; ^2^ Faculty of Mathematics and Information Science, Warsaw University of Technology, Warsaw, Poland; ^3^ Department of Hematology, Institute of Hematology and Transfusiology, Warsaw, Poland; ^4^ Department of Hematology, Medical University Łódź, Łodz, Poland; ^5^ Department of Hematology, Blood Neoplasms and Bone Marrow Transplantation, Wroclaw Medical University, Wrocław, Poland; ^6^ Department of Hematology, Holycross Cancer Center, Kielce, Poland; ^7^ Department of Hematology, Multi-Specialist Hospital Gorzów Wielkopolski, Faculty of Medicine and Health Science, University of Zielona Góra, Gorzów Wielkopolski, Poland; ^8^ Department of Hematology and Transplantology, Medical University of Gdansk, Gdańsk, Poland; ^9^ Department of Hematology, Warmian-Masurian Cancer Center of the Ministry of the Interior and Administration’s Hospital, Olsztyn, Poland; ^10^ Department of Cancer Prevention and Therapy, Wroclaw Medical University, Wrocław, Poland

**Keywords:** azacitidine, infection, myelodysplastic syndromes, acute myeloid leukemia, chronic myelomonocytic leukemia, infection prevention

## Abstract

**Introduction:**

Infections represent one of the most frequent causes of death of higher-risk MDS patients, as reported previously also by our group. Azacitidine Infection Risk Model (AIR), based on red blood cell (RBC) transfusion dependency, neutropenia <0.8 × 10^9^/L, platelet count <50 × 10^9^/L, albumin <35g/L, and ECOG performance status ≥2 has been proposed based on the retrospective data to estimate the risk of infection in azacitidine treated patients.

**Methods:**

The prospective non-intervention study aimed to identify factors predisposing to infection, validate the AIR score, and assess the impact of antimicrobial prophylaxis on the outcome of azacitidine-treated MDS/AML and CMML patients.

**Results:**

We collected data on 307 patients, 57.6 % males, treated with azacitidine: AML (37.8%), MDS (55.0%), and CMML (7.1%). The median age at azacitidine treatment commencement was 71 (range, 18-95) years. 200 (65%) patients were assigned to higher risk AIR group. Antibacterial, antifungal, and antiviral prophylaxis was used in 66.0%, 29.3%, and 25.7% of patients, respectively. In total, 169 infectious episodes (IE) were recorded in 118 (38.4%) patients within the first three azacitidine cycles. In a multivariate analysis ECOG status, RBC transfusion dependency, IPSS-R score, and CRP concentration were statistically significant for infection development (*p* < 0.05). The occurrence of infection within the first three azacitidine cycles was significantly higher in the higher risk AIR group – 47.0% than in lower risk 22.4% (odds ratio (OR) 3.06; 95% CI 1.82-5.30, *p* < 0.05). Administration of antimicrobial prophylaxis did not have a significant impact on all-infection occurrence in multivariate analysis: antibacterial prophylaxis (OR 0.93; 0.41-2.05, *p* = 0.87), antifungal OR 1.24 (0.54-2.85) (*p* = 0.59), antiviral OR 1.24 (0.53-2.82) (*p* = 0.60).

**Discussion:**

The AIR Model effectively discriminates infection-risk patients during azacitidine treatment. Antimicrobial prophylaxis does not decrease the infection rate.

## Introduction

Azacitidine, a hypomethylating agent, is the only drug able to improve overall survival in higher-risk myelodysplastic syndrome (MDS) patients, and hence, it is also the only drug that has been licensed for this indication in Europe since 2010. Following the licensing for MDS, azacitidine has been registered in acute myeloid leukemia (AML) based on its proven efficacy for this indication as well. The incidence of infections in MDS/AML patients treated with azacitidine is very high reaching, especially in real-world practice, up to 62%–75% (1, 2).Infections and transformation to AML represent the main causes of death of higher-risk MDS patients. That is why assessment of the risk of infection is crucial and several studies have aimed to identify factors predisposing to infection, but with divergent results. Recently, the Polish Adult Leukemia Group (PALG), in a retrospective study, has developed a model that stratifies infection risk. The model comprises neutrophil and platelet count, serum albumin level, red blood cell transfusion dependency, and Eastern Cooperative Oncology Group (ECOG) performance status (2). The present study is a prospective validation of the model. It also aims to identify other potential predictive markers.

Antibacterial and antifungal prophylaxis have been shown to reduce the risk of infection- and all-cause-related death incidence in neutropenic patients receiving intensive chemotherapy or allogeneic hematopoietic cell transplantation (allo-HCT) for AML. Therefore, antimicrobial prophylaxis is routinely administered when intensive chemotherapy protocols are used (3). In contrast, no convincing data justifies the administration of antimicrobial prophylaxis in hypomethylating agent-treated patients. Therefore, we aimed to also analyze the impact of antimicrobial agents on infection incidence.

## Patients and methods

### Patient population

Patients diagnosed with MDS, AML, or CMML according to World Health Organization (WHO) 2016 criteria and treated with 5-azacitidine from 1 January 2018 to 31 December 2022 were included and followed up to February 2023. Nine Polish hematology centers aligned with the PALG participated in this prospective non-interventional trial. The study was approved by each Institution’s Ethics Committee in accordance with national legislation and is conducted in accordance with local legal and regulatory requirements and ethical standards including the 1964 Helsinki Declaration and its later amendments to ensure the protection of the patient’s personal data.

### Treatment

Patients received azacitidine dosed initially 75 mg/m^2^ subcutaneously for days 1 through 7 or in a “5–2-2” scheme with a 2-day rest over the weekend. Diagnosis of MDS, AML, or CMML was established based on the WHO 2016 classification (4).

Administration of antibacterial, antiviral, and antifungal prophylaxis was at clinician’s discretion based on center policy. Trimethoprim/sulfamethoxazole, levofloxacin, ciprofloxacin, and occasionally other antibiotics were given for antibacterial prophylaxis, while fluconazole, posaconazole, and voriconazole were prescribed as antifungals, and acyclovir was prescribed as antiviral prophylaxis.

Baseline patient demographics, laboratory data, comorbidities, gut colonization with multidrug-resistant bacteria (MRB), ECOG performance status, MDS-specific Comorbidity Index, BMI (body mass index), cigarette smoking, and transfusion dependency were determined for each patient. The International Prognostic Scoring System (IPSS) and IPSS-revised (IPSS-R) were applied to MDS, AML with 20%–30% marrow blasts, and CMML patients (5–7).

An infectious episode was defined as Grade ≥ III infection according to the Common Terminology Criteria for Adverse Events version 3.0—neutrophil count threshold was decreased from 1.0 × 10^9^/L to 0.5 × 10^9^/L for neutropenic fever; consequently, in our analyses, neutropenic fever was defined as neutropenia 38°C persisting for more than 1 h, or body temperature 38.3°C recorded on a single occasion (Freifeld 2010). Invasive fungal infections were defined according to European Organization for Research and Treatment of Cancer (EORTC)/National Institute of Allergy and Infectious Diseases Mycoses Study Group criteria (Pauw 2010). Infectious episodes were classified as microbiologically defined (if positive microbiological samples were available) and/or clinically defined (physician’s judgment based on clinical course, lab tests, imaging, and treatment outcome).

The Azacitidine Infectious Risk Model described in ACLM 2019 was applied to stratify patients. RBC transfusion dependency, neutropenia < 0.8 × 10^9^/L, platelet count < 50 × 10^9^/L, albumin level < 35 g/dL, and ECOG performance status ≥ 2 were assigned one point each, and the infection rate was finally determined for each total score grouping patients into two categories: lower (0–2 score) and higher early infection risk (3–5 score).

### Statistical analysis

All analyses were conducted using statistical software R version 4.3 (R Foundation for Statistical Computing, Vienna, Austria). Survival curves were calculated with the Kaplan–Meier estimator, and hazard ratios were calculated with Cox proportional hazards model. Median survival was calculated based on Kaplan–Meier curves. *p*-values for group comparisons (**Table 1**) were calculated with Pearson’s *χ* ^2^ test for categorical variables and Wilcoxon test for continuous variables. The predictive model was built using a two-step procedure. In the first step (variable filtering), pairwise relations between infections and other variables were calculated. Then, the most significant variables were converted into binary variables. Based on these variables, the predictive model was built using the logistic regression method.

**Table 1:** Patients’ and azacitidine treatment characteristics.

Characteristics	n (%)
Sex	
Male	177 (57.6%)
Female	130 (42.4%)
Age, median (range); years	71 (18–95)
WHO 2016 diagnosis	
AML	116 (37.8%)
MDS	169 (55.0%)
CMML	22 (7.1%)
IPSS	
Low	1 (0.3%)
Int 1	16 (5.2%)
Int 2	102 (33.2%)
High	89 (28.9%)
NA	99 (33.2%)
IPSS-R	
Very Low	0 (0.0%)
Low	2 (0.6%)
Intermediate	21 (6.8%)
High	75 (24.4%)
Very High	103 (33.5%)
NA	106 (34.5%)
ECOG Performance Status	
0–1	213 (69.3%)
2–4	94 (30.7%)
MDS Comorbidity Index	
0-1 2-3	231 (75.4%) 76 (24.6%)
BMI	
<25 kg/m^2^ ≥ 25 kg/m^2^ No data	107 (34.8%) 161 (52.4%) 39 (12.8%)
Transfusion dependency	
RBC TD	168 (54.7%)
PLT TD	71 (23.1%)
Comorbidities	
DM	67 (21.8%)
COPD	22 (7.1%))
Cardiac failure (NYHA III/IV)	40 (13.2%)
Second malignancy	68 (22.1%)
Corticosteroid treatment	18 (20.4)
Autoimmune disease	41 (13.3%)
Tobacco smokers	86 (28.0%)
MRB colonization	50 (16.2%)
Antimicrobial Prophylaxis	
Antibacterial	205 (66.0%)
Antifungal Antiviral	90 (29.3%) 79 (25.7%)
5-AZA administration	
Inpatient	243 (79.2%)
Outpatient	64 (20.8%)

WHO, World Health Organization; AML, acute myeloid leukemia; MDS, myelodysplastic syndromes; CMML, chronic myelomonocytic leukemia; ECOG, Eastern Cooperative Oncology Group; IPSS, international prognostic scoring system; IPSS-R, revised international prognostic scoring system; MDS, myelodysplastic syndrome; RBC TD, Red Blood Cell Transfusion Dependence; PLT TD, Platelet Transfusion Dependence; NYHA, New York Heart Association; DM, Diabetes mellitus; COPD, Chronic obstructive pulmonary disease; MRB, multidrug-resistant bacteria; 5-AZA, azacitidine.

## Results

### Baseline patient characteristics

We collected data on 307 patients treated with azacitidine: AML, *n* = 116 (37.8%); MDS, *n* = 169 (55.0%); and CMML, *n* = 22 (7.1%). A total of 231 patients received at least three azacitidine cycles. Median number of administered cycles was 4 (range, 4–5). The median age at azacitidine commencement was 71 (range, 18–95) years and men predominated (*n* = 177; 57.6%) in the analyzed group. IPSS-R was calculated for 201 (65.5%) patients, and most of them belonged to high-risk (37.3%) or very-high-risk (51.2%) groups. Gut MRB colonization was found in 55 (16.2%) patients before azacitidine treatment and in 55 (17.9%) during the first three azacitidine cycles. Antibacterial, antifungal, and antiviral prophylaxes were used in 66.0%, 29.3%, and in 25.7% patients, respectively. Detailed information on baseline patients’ characteristics is presented in **Table 1**.

### Infection characteristics

In total, 169 infectious episodes (IEs) were recorded in 118 (38.4%) patients during the first three azacitidine cycles. Clinical characteristic is presented in **Table 2**. Most of them [90 IEs (50.0%)] were classified as bacterial, 19 IEs (10.6%) were classified as fungal, and 15 IEs (8.3%) were classified as viral, and 56 IEs (31.1%) were not microbiologically categorized (**Table 3**). Some episodes were classified by more information simultaneously (e.g., clinical presentation and microbiological characterizations), resulting in 198 clinically characterized and 180 microbiologically classified infections. Two infections were recorded in 27 patients and at least three IEs in 10 patients. Septic shock occurred in 11 IEs (5.5%).

**Table 2:** Infection characteristics.

Infection category	% of infectious episodes
Neutropenic fever	13.1
FUO (without neutropenia)	3.0
Pneumonia	40.4
Sepsis (including septic shock)	8.5
Septic shock	5.5
Gastrointestinal tract infection	9.1
Skin and soft tissue infection	5.6
Urinary tract infection	8.1
Other	6.6

FUO, fever of unknown origin.

**Table 3:** Microbiologically confirmed infection.

Type of infection		(n)	Pathogen	(n)
Bacterial		58		
			Gram–positive bacteria	32
			Coagulase-negative *Staphylococcus*	7
			*Staphylococcus aureus*	6
			*Enterococcus* spp.	8
			*VRE* *Clostridioides difficile* *Streptococcus agalactiae*	5 10 1
			Gram-negative bacteria	26
			* Escherichia coli* * ESBL*	8 4
			* Klebsiella pneumoniae* * ESBL* * MBL* *Citrobacter freundi*	9 6 3 1
			*Morganella morganii*	1
			*Pseudomonas aeruginosa*	5
			*Stenotrophomonas maltophilia*	1
			*Mycobacterium tuberculosis*	1
Fungal		10		
	Proven	7	*Aspergillus fumigatus*	2
			*Candida albicans* *Candida glabrata* *Mucor*	3 1 1
	Probable	3		
Viral		13		
			*Influenza A* *SARS-CoV2*	1 12

VRE, Vancomycin-Resistant Enterococcus; ESBL, extended-spectrum beta-lactamases; MBL, metallo-beta-lactama; SARS-CoV2, severe acute respiratory syndrome coronavirus 2.

### Infectious risk

Univariate analysis showed that ECOG status, cytogenetics IPSS R score, marrow blast percentage, ferritin, IgG and CRP concentration, red blood cell, platelet transfusion dependency, and serum albumin level were associated with the risk of infection (for details, please see **Table 4**).

**Table 4:** Univariate analysis of risk factors for infection within the first 3 cycles of azacitidine treatment.

Characteristics	With infection in the first 3 cycles N (%) or median (range)	Without infection in the first 3 cycles N (%) or median (range)	p-value
Sex			
Male	66 (55.9)	111 (58.7)	0.716
Female	52 (44.1)	78 (41.2)	
Age, median, range	72 (33–88)	71 (18–95)	0.251
WHO 2016 diagnosis			0.024*
AML	52 (44.0)	64 (33.8)	
MDS	54 (45.7)	115 (60.8)	
CMML	12 (10.1)	10 (5.2)	
IPSS			0.22
Low	0 (0.0)	1 (0.5)	
Int 1	5 (4.2)	11 (5.8)	
Int 2	36 (30.5)	66 (34.9)	
High	39 (33.0)	50 (26.4)	
NA	38 (32.2)	61 (32.2)	
IPSS-R			0.21
Very Low	0 (0.0)	0 (0.0)	
Low	2 (0.8)	1 (0.5)	
Intermediate	7 (5.9)	14 (7.4)	
High	23 (19.5)	52 (27.5)	
Very High	47 (39.8)	56 (29.6)	
NA	40 (33.9)	66 (34.9)	
ECOG Performance Status			0+
0–1	61 (51.6)	152 (80.4)	
2–4	57 (48.3)	37 (19.5)	
IPSS cytogenetics			0.055
Low	38 (32.2)	70 (37.0)	
Intermediate	18 (15.2)	40 (21.1)	
High	20 (16.9)	28 (14.8)	
NA	42 (35.6)	51 (26.9)	
IPSS-R cytogenetics			0.031*
Very Good	38 (32.2)	70 (37.0)	
Good	1 (0.8)	3 (1.5)	
Intermediate	19 (16.1)	45 (23.8)	
Poor/Very Poor	25 (21.1)	35 (18.5)	
NA	35 (29.6)	36 (19.0)	
Vit D3	18.0 (7.2-40)	20.0 (8.0-80.1)	0.179
RBC TD	82 (70.0)	86 (46.0)	0+
Diabetes mellitus	25 (21.5)	42 (22.5)	0.946
COPD	13 (11.2)	9 (4.8)	0.067
Cardiac failure (NYHA III/IV)	21 (18.1)	19 (10.1)	0.070
Second malignancy	24 (20.6)	44 (23.4)	0.681
Corticosteroid treatment	10 (8.5)	10 (5.3)	0.384
Autoimmune disease	16 (13.9)	25 (13.2)	1
CRP (mg/L)	20.5 (0-242)	6 (0-200)	0+
Hemoglobin (g/dl)	8.9 (4.7–97.0)	9.0 (4.5–91.5)	0.172
Platelets (× 10^9^/l)	45 (4–828)	61 (5–628)	0.162
Neutrophils (× 10^9^/l)	0.63 (0.0–56.8)	0.87 (0.02–39.0)	0.143
Lymphocytes (× 10^9^/l)	1.16 (0.0–9.5)	1.30 (0.1–11.0)	0.516
Monocytes (× 10^9^/l)	0.35 (0.0–41.0)	0.30 (0.0–34.5)	0.837
Bone marrow blasts(%)	16.7 (1.0–96.2)	14.0 (0.0–93.0)	0.016
IgG (g/l)	1121 (100-3937)	900(100–1180)	0.026*
Ferritin (ng/ml)	1144 (61–11099)	574 (11–7932)	0.001*
Iron (g/dl)	121 (27–267)	124 (6.8–291)	0.428
Albumin (g/dl)	3.6 (1.9–5.4)	3.9 (2.6–9.97)	0.0004*

AML, acute myeloid leukemia; CMML, chronic myelomonocytic leukemia; CRP, C-reactive protein; ECOG, Eastern Cooperative Oncology Group; IgG, immunoglobulin G; Int, intermediate; IPSS, international prognostic scoring system; IPSS-R, revised international prognostic; scoring system; MDS, myelodysplastic syndrome; NA, not available; NYHA, New York Heart Association; RBC TD, Red Blood Cell Transfusion Dependence; COPD, Chronic obstructive pulmonary disease.

* - statistically significant meaning p value < 0,05.

In a multivariate analysis, only ECOG status, RBC transfusion dependency, IPSS-R score, and CRP concentration retained significance (**Table 5**).

**Table 5:** Multivariate generalized mixed model (logistic), odds of getting infection in any cycle.

Parameter	Cutoff	Odds Ratio	95% confidence interval	Significance P-value
CRP	> 20 mg/L	2.68	1.39–5.19	0.003*
ECOG	> 2	2.73	1.42–5.31	0.002*
WBC	> 6.5 x10^9^/L	2.27	1.07-4.73	0.03*
RBC TD	Yes	3.07	1.37–7.85	0.01*
R-IPSS	> 2	2.37	1.23–4.68	0.01*

CRP, C-reactive protein; ECOG, Eastern Cooperative Oncology Group; IPSS-R, revised international prognostic; WBC, White Blood Cells; RBC TD, Red blood cells transfusion dependency.

* - statistically significant meaning p value < 0,05.

Most patients [200 (65%)] were assigned to the higher-risk AIR Model group. The occurrence of infection within the first three azacitidine cycles was significantly higher in the higher-risk group (47.0%) than in the lower-risk group (22.4%) [odds ratio 3.06 (1.82–5.30) (*p* < 0.05)]. The sensitivity of the model was 80, and specificity was 44.

### Antimicrobial prophylaxis

In most patients (224, 73%), antimicrobial prophylaxis was administered during the first three azacitidine cycles. Infections occurred more frequently in patients with prophylaxis (44%) than in patients without prophylaxis (22%) (*p* = 0.001) during any of the first three AZA cycles; the difference was significant in the first cycle but it disappeared in the second and the third cycle, if analyzed separately (**Table 6**).

**Table 6:** Relative frequency of infection in patients with and without prophylaxis.

Infection	No prophylaxis (n-72)	Prophylaxis (n-197)	p-value
**Infection during any of the first 3 cycles (total)**	22%	44%	0.001*
**Infection** **1-st cycle**	13.8%	29.1%	0.001*
**Infection** **2-nd cycle**	16.2%	24.3%	0.14
**Infection** **3-rd cycle**	10.1%	13.3%	0.6

* - statistically significant meaning p value < 0,05.

The cohorts with prophylaxis vs. no prophylaxis differed significantly in terms of (1) underlying diagnosis—more frequent administration of prophylaxis in AML (62%) than in MDS (43%) or CMML (36%), (2) previous treatment—more prevalent in patients exposed previously to chemotherapy (62.9% vs. 46.3%), (3) prevalence of neutropenia (neutropenia below 0.8 × 10^9^/L present in 57.6% vs. 40.6%), and (4) platelet transfusion dependency (60.8% vs. 46.0% in non-platelet transfused patients).

Administration of antimicrobial prophylaxis did not have a significant impact on all-infection occurrence in multivariate analysis: antibacterial prophylaxis OR 0.93 (0.41–2.05), *p* = 0.87; antifungal OR 1.24 (0.54–2.85), *p* = 0.59; and antiviral OR 1.24 (0.53–2.82), *p* = 0.60.

### Clinical outcome

Median follow-up was 16 months (95% CI, 13–18) and 214 patients (70.0%) died during that time. Median overall survival was 11 months (95% CI, 9–14).

**Figure 1:**
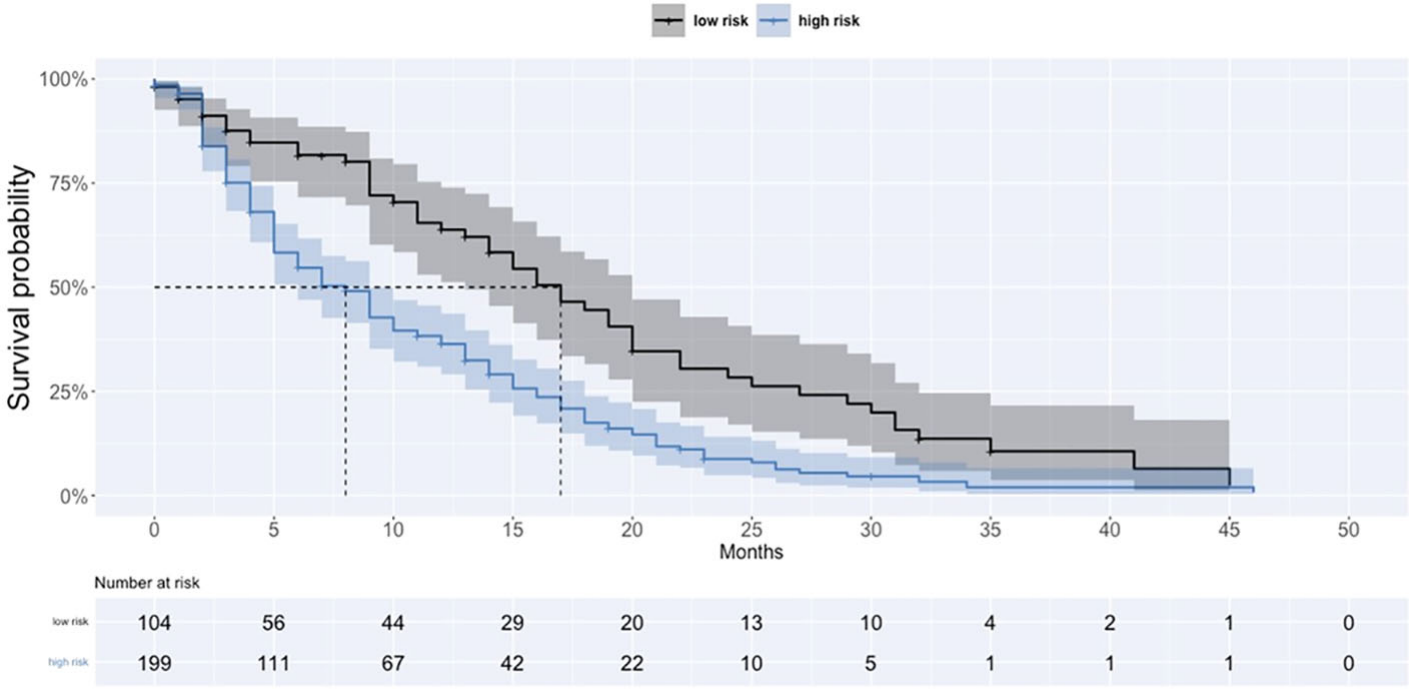
Survival curves in high and low infection risk according to AIR group.

The median overall survival in the higher-risk group was 9 months (6–10), while that in the lower-risk group is 19 months (15–24) (**Figure 1**). Fatal outcome was recorded in 25 out of 169 IEs (14.7%). Infection-related attributable mortality was 8.1% (25/307) in the whole group and 21.5% (25/118) in patients who developed infections. The median survival in patients with infection was 6 months (5–9), while that in patients without infection was 15 months (12–17).

## Discussion

Azacitidine is associated with an overall survival advantage in comparison to other regimens in AML/MDS/CMML patients unfit for intensive chemotherapy and allogeneic hematopoietic cell transplantation (8, 9). Its favorable effect is probably counterbalanced by the risk of development of serious infections. The incidence of infectious episodes in MDS patients is significantly increased when compared to the age-matched general population (10).It remains the subject of debate if administration of hypomethylating agents (HMAs)/azacitidine *per se* is associated with grade III/IV infection. In Shargian-Alon et al.’s meta-analysis covering nine randomized controlled trials comparing HMA-containing regimens with other regimens administered to MDS/AML patients, it was found that HMA treatment increases the risk of serious infection. However, the comparator group was very divergent in the studies, including best supportive care. It is worth noting that all-cause mortality was reduced in the HMA group (11).

In our study almost 40% of patients developed grade III/IV infection during the first three azacitidine cycles. It is in line with previous reports where also most infections occurred within the first three azacitidine cycles (12, 13). Prediction of infection is crucial in patients treated with azacitidine. In our cohort ECOG status, RBC transfusion dependency, IPSS R-score, and CRP concentration were significant predictive factors in multivariate analysis. Previously, our group developed the Azacitidine Infection Risk (AIR) Model including ECOG performance status, platelet and neutrophil count, albumin serum concentration, and RBC transfusion dependency with a sensitivity of 62% and a specificity of 86% (Madry et al.) (2). In the present study, albumin concentration retained its significance only in univariate analysis, while patients with lower neutrophil and platelet count had a tendency for higher infection occurrence, but the difference was insignificant. Nonetheless, using the combination of factors applied in the AIR Model, we confirmed the validity of this model in the current analysis with a sensitivity of 80% and a specificity of 44%. Two times more patients (47%) suffered from infection in the higher-risk group in comparison with the lower-risk group (22%). Patients assigned to the higher-risk group lived significantly shorter, with a median OS of 9 months vs. 19 months.

Serum concentration of C-reactive protein is commonly used as an infection marker but with an undefined practical role according to guidelines addressed to patients with hematologic malignancies and febrile neutropenia (14). In our analysis, elevated CRP > 20 mg/L before the commencement of AZA was found to be an independent predictor of infection in patients with no other clinical manifestation of infection. In the Sugiura et al. study, asymptomatic CRP elevation anteceded febrile neutropenia occurrence in 55.6% of cases, and prophylactic application of broad-spectrum antibiotics in patients with elevated CRP reduced infection rates from 31% to 6.7% (15). It is interesting to note that the ratio of CRP to albumin was found to be associated with worse response rate and shorter overall survival in newly diagnosed AML patients (16).

Our patients were relatively well microbiologically characterized with available microbiological results in 45% of cases in comparison to other studies, where such a confirmation was documented only in 15%–54% of infectious episodes (12, 17).Pneumonia was the most frequent clinical presentation (40%), with the rate being much higher than in other studies (16%–30%). It may probably be attributable to the SARS-CoV-2 pandemic era (11 cases with COVID-19 pneumonia).

In our study, 73% of patients received any antimicrobial prophylaxis in contrast to the lower rate in previous studies (31%–49%) (1, 18). There is no clear explanation for this difference; the proportion of neutropenic and AML patients included were not significantly different. Decision on prophylaxis administration remained at the physician’s discretion and perhaps was also the result of environmental factors and the COVID-19 pandemic. It may be hypothesized that the treating physicians could have tried to prevent the occurrence of infection by any means to keep the patient alive and safe off the hospital, which, especially at the beginning of the pandemic, could become the site of the SARS-CoV-2 spread.

In our work, infectious complications were observed more commonly in patients taking prophylaxis (44% vs. 22%), especially during the first cycle. Nonetheless, in multivariate analysis, having received antibacterial prophylaxis did not retain a significant impact on infection occurrence probably due to the imbalance between the groups, i.e., with prophylaxis and without prophylaxis. Patients with prophylaxis belonged to the potentially higher-risk groups with more AML patients, with more severe/profound neutropenia and with azacitidine used as a subsequent antineoplastic therapy.

Antibiotic prophylaxis was not routinely used in available randomized clinical trials of MDS/AML patients treated with hypomethylating agents; therefore, it was not possible to assess its impact on infection risk (11). Data on antimicrobial prophylaxis derived from retrospective studies reported divergent results. Some of them demonstrated reduced bacterial infection incidence in patients receiving antimicrobial prophylaxis especially in neutropenic patients (neutrophil count < 0.5 × 10^9^/L), while others did not show any impact (1, 13, 18–21).

Our study has an advantage over other analyses in terms of its prospective design. Nevertheless, it is also not randomized and does not control for intervariabiliity between groups in terms of population and intervention choice.

Antibacterial prophylaxis is routinely used in AML patients treated with intensive chemotherapy. Fluoroquinolone prophylaxis is associated with a lower rate of bloodstream infections and episodes of fever during neutropenia, although recently its positive effect on reducing mortality has been contested (22). Different AML and MDS biology and intensive chemotherapy (IC) and HMA mechanisms of action do not allow translating conclusions regarding prophylaxis in IC-treated patients into HMA management. MDS patients are characterized by prolonged neutropenia and delayed response to HMA; thus, long-term antibiotic treatment may induce resistance, adverse events, and the development of MDR bacterial gut colonization. There are reports demonstrating that prolonged quinolone administration significantly increases antimicrobial resistance, especially among Gram-negative bacteria, not only to quinolones but also to piperacilin/tazobactam and carbapenems (23, 24).

As mentioned earlier, our study has some limitations including the heterogeneous population of patients enrolled in regard to the underlying diagnosis (AML, CMML, and MDS), azacitidine used in different lines of therapy, and non-randomized, diverse antimicrobial prophylactic management. Another limitation is that, since 2021, the venetoclax/azacitidine (Ven/AZA) combination therapy has been the standard of care for unfit AML patients and the number of azacitidine monotherapy-treated AML patients in routine practice is limited nowadays. However, our study has been designed and initiated in 2018 before Ven/AZA was licensed in AML. We decided against the co-option of Ven/AZA-treated patients from 2021 because it would harm the group coherence and may further complicate the interpretation of the results.

On the other hand, this is one of the largest studies focusing on infection in azacitidine treated patients reflecting real-life of prospectively included and in detail clinically and microbiologically described MDS/AML/CMML population.

## Data availability statement

The original contributions presented in the study are included in the article/supplementary material. Further inquiries can be directed to the corresponding author.

## Ethics statement

The studies involving humans were approved by Bioethics Committee, Medical University Warsaw. The studies were conducted in accordance with the local legislation and institutional requirements. The participants provided their written informed consent to participate in this study.

## Author contributions

KM: Conceptualization, Data curation, Formal analysis, Investigation, Methodology, Project administration, Supervision, Validation, Visualization, Writing – original draft, Writing – review & editing. KL: Conceptualization, Data curation, Formal analysis, Investigation, Methodology, Project administration, Supervision, Validation, Visualization, Writing – original draft, Writing – review & editing. ESi: Conceptualization, Data curation, Formal analysis, Methodology, Project administration, Supervision, Validation, Visualization, Writing – original draft, Writing – review & editing. JD-S: Conceptualization, Data curation, Formal analysis, Investigation, Methodology, Supervision, Writing – original draft, Writing – review & editing. PB: Conceptualization, Data curation, Formal analysis, Investigation, Methodology, Project administration, Supervision, Writing – original draft, Writing – review & editing. OS: Conceptualization, Data curation, Formal analysis, Investigation, Methodology, Project administration, Supervision, Validation, Writing – original draft, Writing – review & editing. AG: Data curation, Investigation, Methodology, Writing – original draft, Writing – review & editing. MO-S: Conceptualization, Data curation, Formal analysis, Investigation, Methodology, Project administration, Supervision, Validation, Writing – original draft, Writing – review & editing. AO: Conceptualization, Data curation, Formal analysis, Investigation, Methodology, Supervision, Writing – original draft, Writing – review & editing. ZW: Conceptualization, Data curation, Formal analysis, Investigation, Methodology, Project administration, Supervision, Validation, Writing – original draft, Writing – review & editing. JŚ: Conceptualization, Data curation, Formal analysis, Investigation, Methodology, Project administration, Supervision, Validation, Writing – original draft, Writing – review & editing. ESu: Conceptualization, Data curation, Formal analysis, Investigation, Methodology, Project administration, Supervision, Validation, Writing – original draft, Writing – review & editing. AB: Conceptualization, Data curation, Formal analysis, Investigation, Methodology, Project administration, Supervision, Validation, Writing – original draft, Writing – review & editing. RM: Conceptualization, Data curation, Formal analysis, Investigation, Methodology, Project administration, Software, Supervision, Validation, Visualization, Writing – original draft, Writing – review & editing. KB: Conceptualization, Data curation, Formal analysis, Investigation, Methodology, Project administration, Supervision, Validation, Writing – original draft, Writing – review & editing. GB: Conceptualization, Data curation, Formal analysis, Investigation, Methodology, Project administration, Supervision, Validation, Writing – original draft, Writing – review & editing.
